# HIV Pre-Exposure Prophylaxis (PrEP) Counseling in Germany: Knowledge, Attitudes and Practice in Non-governmental and in Public HIV and STI Testing and Counseling Centers

**DOI:** 10.3389/fpubh.2020.00298

**Published:** 2020-07-14

**Authors:** Frank Kutscha, Matthew Gaskins, Mary Sammons, Alexander Nast, Ricardo Niklas Werner

**Affiliations:** ^1^Schwulenberatung Berlin, Berlin, Germany; ^2^Alice Salomon Hochschule Berlin—University of Applied Sciences, Berlin, Germany; ^3^Division of Evidence-Based Medicine (dEBM), Department of Dermatology, Venerology and Allergy, Charité-Universitätsmedizin Berlin, Corporate Member of Freie Universität Berlin, Humboldt-Universität zu Berlin and Berlin Institute of Health, Berlin, Germany

**Keywords:** HIV pre-exposure prophylaxis, PrEP, counseling, public health, HIV prevention, health services research

## Abstract

**Background:** Providers of sexual health services play an important role in counseling persons at risk of acquiring HIV. The aim of the present study was to investigate the knowledge of and attitudes toward HIV pre-exposure prophylaxis (PrEP) among counselors in non-governmental counseling centers (“NG counseling centers”) and in counseling centers of the local health authorities (“local health offices”) in Germany and to determine the extent to which PrEP plays a role in their current counseling practice.

**Methods:** An anonymous cross-sectional study using an online questionnaire was conducted among counselors from sexual health centers across Germany. All NG counseling centers in Germany offering HIV testing were asked to participate. For each NG counseling center, a local health office in the same city was also invited. A “knowledge score” and an “attitudes score” were calculated from single items on various relevant aspects. The association of these scores with the proportion of PrEP advice provided proactively in sessions with men who have sex with men (MSM) and trans persons who met the German and Austrian guideline criteria for being offered PrEP (‘at-risk clients’) was quantified.

**Results:** From Oct. to Dec. 2018, 145 counselors completed the survey. Both self-assessed knowledge of PrEP and attitudes toward PrEP were greater or more positive among counselors from NG counseling centers compared with counselors from local health offices [*Median* knowledge score (range 0-20): 18.0 (*IQR* = 5.0) vs. 14.0 (*IQR* = 4.0), *p* < 0.001; *median* attitudes score (range 0-20): 18.0 (*IQR* = 4.0) vs. 14.0 (*IQR* = 6.8), *p* < 0.001]. The proportion of PrEP advice provided proactively in sessions with at-risk clients was larger in counseling centers than in local health offices [50.0% (*IQR* = 60.0) vs. 30.0% (*IQR* = 70.0); *p* = 0.003]. The results of the multiple linear regression model indicate that knowledge and attitudes of the individual counselors, but not the type of center in which they worked, were independent predictors of the proportion of proactive advice on PrEP.

**Conclusions:** There is room for improvement in the current PrEP counseling practice of sexual health services in Germany. The findings of the present study suggest opportunities to improve the implementation of PrEP as part of a comprehensive HIV prevention strategy.

## Introduction

In 2018, an estimated 2,400 new infections with HIV, primarily in men who have sex with men (MSM), occurred in Germany (1) and the prevention of HIV remains a major public health concern (2). Public health strategies to prevent sexually transmitted HIV infection have traditionally focused on behavioral interventions such as supporting condom use in sexually active populations. However, apart from the effects of early diagnosis and treatment of HIV infections, the latter of which is highly effective at preventing the transmission of HIV (3–5), a relatively new form of biomedical HIV prevention, namely HIV pre-exposure prophylaxis (PrEP), and its broad implementation in populations at risk has likely contributed to declining HIV incidence rates in some major cities such as San Francisco, London, and Sydney (6–8). The efficacy and safety of PrEP in MSM and trans persons has been shown in various randomized controlled studies (9–13) and cohort studies (14–17). International and national guidelines recommend the use of PrEP for HIV-negative people at substantial risk of acquiring HIV (18–20). According to the German and Austrian guideline published in May 2018, PrEP should be offered to HIV-negative adult MSM and trans persons who had a sexually transmitted infection (STI) in the past 12 months or who report having had and/or having the intent to practice condom-less anal sex (19). The cost of PrEP has been covered by the public health insurance in Germany only since September 2019.

In the US, the uptake of PrEP has been influenced largely by different, primarily socioeconomic and individual information-related factors (21–27). For the German context, few data on PrEP use are available. A survey among MSM in Berlin identified a substantial gap between the indication to use PrEP and actual PrEP use: almost a quarter of the non-PrEP-using participants reported sexual behavior that put them at risk of acquiring HIV (28). Results from a survey among users of a dating platform for MSM in 2018 suggest that PrEP use among MSM in Germany is less common compared with some other western European countries (29). Access to information about PrEP has been shown to be an important barrier to the initiation of PrEP (28). However, surveys from the US suggest that knowledge of PrEP may also be limited among physicians (30, 31). A survey among Dutch providers of STI and HIV services showed a moderate willingness to prescribe PrEP and limited knowledge of PrEP, especially among STI specialists (32). The provision of PrEP-related training for physicians has been discussed as a factor that could improve the implementation of PrEP (30, 31).

In Germany, HIV testing and counseling on HIV prevention is provided primarily by specialist physicians working in office-based practices and by sexual health services such as HIV and STI testing and counseling centers. The latter are either non-governmental, community-based counseling centers (“NG counseling centers”) or public sexual health services of the local health authorities (“local health offices”). Both types of counseling centers offer low-threshold, anonymous HIV and STI testing and counseling and play an important role in the dissemination of information on HIV and strategies to prevent infections (2, 33). To date, no data have been available on PrEP-related counseling competence, knowledge, and attitudes among counselors working at either type of organization.

With the present study, we aimed to investigate the extent to which PrEP plays a role in the counselors' counseling practice, as well as the factors influencing the proportion of proactive PrEP advice they provide to clients at risk of sexually acquired HIV. Furthermore, the relevance of different barriers for potential PrEP users to initiate PrEP as perceived by the counselors was evaluated. With this knowledge we aimed to identify barriers to and facilitators of PrEP implementation and opportunities to improve PrEP implementation among MSM and trans persons in Germany. This study comprised part of the master's thesis of the first author (FK).

## Methods

### Study Design

For this cross-sectional study, an online survey was conducted among counselors working in non-governmental, community-based counseling centers (“NG counseling centers”) or in counseling centers of the local health authorities (“local health offices”). The anonymous, self-administered questionnaire was available online from October to December 2018. The study protocol was approved by the local ethics committee of Charité – Universitätsmedizin Berlin (EA1/006/19) and informed consent was obtained from all participants.

### Setting and Eligibility

Counselors from NG counseling centers and local health offices were eligible to participate if they worked in the field of counseling clients on HIV and other STIs, regardless of their primary professional qualification. All NG counseling centers offering HIV testing and counseling listed by the comprehensive, publicly available register provided by “HIV and more” (34) were asked to participate. In order to ensure the comparability of NG counseling centers with the participating local health offices, a local health office in the same or in a comparable city was invited for each NG counseling center. The selected centers were contacted by email and requested to forward the survey invitation to all eligible counselors within their organization. A reminder email was sent three to four weeks after the initial invitation. Additionally, all centers were contacted by telephone to enhance the participation rate. This telephone call also served to obtain information on the number of counselors to whom the invitation email had been forwarded in each organization.

### Questionnaire and Variables

A standardized German-language questionnaire exploring knowledge of and attitudes toward PrEP among counselors and their counseling practice on PrEP is not available; the questionnaire was therefore developed for the purpose of the present study. The original draft questionnaire (FK) was tested and discussed (RW, MG, MS, AN) to identify and correct errors concerning spelling, expression and grammar as well as problems concerning the comprehensibility of the content and design. The questionnaire covered the following topics:

- Socio-demographic data and information about the type of center
- Counseling sessions and counseling practice regarding PrEP
- Self-assessed PrEP-related knowledge and self-reported attitudes
- Need for information or training materials to improve PrEP counseling
- Perceived barriers for potential users to initiate PrEP.

After providing informed consent and answering the initial question on the type of center (NG counseling center vs. local health office), participants could, if they so desired, leave any number of questions unanswered. The participants were provided with a brief summary on the efficacy and safety of PrEP, and with information about the recommendations of the German and Austrian guideline on the indications for offering PrEP.

Socio-demographic data comprised gender, age, primary professional qualification and work experience in counseling on sexual health issues. Furthermore, contextual information on the counseling center was obtained, including which of the 16 states in Germany the organization was located and size of the city, the number of HIV tests provided per month and the number of these that were positive. In addition, the total number of personal counseling sessions with MSM and trans persons per month and the number of sessions with MSM and trans persons who met the criteria to be counseled on PrEP according to the German and Austrian guideline (“at-risk clients”), was obtained. Regarding counseling practice, participants were asked to indicate the proportion of counseling sessions with these at-risk-clients (a) in which the topic PrEP had been addressed by the clients themselves and (b) in which the counselors themselves had proactively addressed the topic.

PrEP-related knowledge and counseling competence and the attitudes toward PrEP were quantified using a self-assessment in terms of agreement with statements about various aspects relevant in this context (Table 1). Fully verbalized bipolar five-step Likert scales with an ambivalent scale center were provided to quantify the agreement with these statements. The items were presented randomly to each participant in a different order. Since the evaluation of individual Likert-scaled variables is considered less reliable compared to a summative multi-item scale (35), a summative “knowledge score” and “attitudes score” were calculated from the five individual knowledge and attitudes variables. The total scores assume values between 0 and 20, with high values representing good knowledge and counseling competence, or positive attitudes toward PrEP, respectively.

**Table 1:** Dimensions and their operationalisation used to assess **(A)** knowledge and counseling competence and **(B)** attitudes toward PrEP.

**Dimension**		**Operationalisation and scores**				
***Do you agree or disagree with the following statements?***		***Strongly disagree***	***Disagree***	***Neither agree nor disagree***	***Agree***	***Strongly agree***
**(A) Knowledge and counseling competence**						
Global assessment	“I am well-informed about PrEP”	0	1	2	3	4
Indications	“I am able to comprehensively give clients advice on whether it makes sense to take PrEP in their respective case”	0	1	2	3	4
Adverse effects	“I am able to comprehensively give clients advice on the adverse effects of PrEP”	0	1	2	3	4
Modalities of intake	“I am able to comprehensively give clients advice on the possible modalities of intake of PrEP (e.g., continuous vs. on-demand)”	0	1	2	3	4
Investigations	“I am able to comprehensively give clients advice on the medical investigations necessary during the use of PrEP”	0	1	2	3	4
‘Knowledge score’		Summative score with values ranging from 0 to 20				
**(B) Attitudes toward PrEP**						
Global assessment	“I think that PrEP is an important element of HIV prevention strategies”	0	1	2	3	4
Reliability	“I think that PrEP is a reliable method to protect oneself from HIV”	0	1	2	3	4
Adverse effects	“I think that PrEP is a method to protect oneself from HIV that has few side effects”	0	1	2	3	4
Availability of better alternatives	“I think that PrEP is unnecessary, because there are better alternatives to protect oneself from HIV”	4	3	2	1	0
Reimbursement of costs	“I think that PrEP should be paid for by the statutory health insurance”	0	1	2	3	4
‘Attitudes score’		Summative score with values ranging from 0 to 20				

Furthermore, the participants were asked whether training was offered and whether inhouse guidelines or standard operating procedures on PrEP counseling were available in their organization. In addition, participants could indicate whether they wished to receive training or information material on PrEP counseling. A multiple-choice list was offered to assess tools or training that could be helpful to improve their counseling work or enhance practicability. To assess potential barriers to PrEP initiation, various aspects were presented, and participants were asked to rate the relevance of these potential barriers on an eleven-level, end-verbalized rating scale with numeric markers (0 = no relevance to 10 = highest relevance) according to their counseling experience. Again, the items were presented to each participant in randomized order.

### Sample Size and Statistical Methods

Since the questionnaire was expressly designed for the purpose of this study, no data on expected means and variability were available. The aim was to include all NG counseling centers offering HIV testing and a corresponding number of matched local health offices. Therefore, no sample size calculation was performed. Statistical analyses were conducted with IBM^®^ SPSS^®^ Statistics version 25 (sample characteristics and bivariate statistics) and with STATA SE version 14.2 (multiple linear regression). To describe the sample characteristics and the results, descriptive statistics were used depending on the data quality. The internal consistency of the summative knowledge and attitudes scores was quantified with Crohnbach's alpha. To quantify associations between variables, independent samples *t*-tests, the Mann-Whitney *U* test and Pearson's Chi squared tests were used, depending on the data quality. A multiple linear regression, using the backward elimination method, was modeled to identify predictors of the proportion of proactive advice on PrEP in sessions with at-risk clients. The following variables for this regression model were purposefully selected a priori: type of center (NG counseling center vs. local health office), gender and years of practical work experience of the participant, size of the city, rate of positive HIV tests, knowledge score, and attitudes score. The stopping rule for the elimination of individual variables in the multiple linear regression was *p* < 0.2. Variance inflation factor (VIF) statistics and condition number were used to verify that there was no multi-collinearity of the predictors and instability of the regression coefficients, respectively. Missing cases were excluded in a listwise fashion. The level for statistical significance was set at *p* = 0.05.

## Results

### Recruitment, Participation, and Response Rate

The letter of invitation to participate in the survey was sent to a total of 76 centers (38 NG counseling centers and 38 local health offices). Overall, 179 counselors opened the survey and began to fill it in. Of these, 145 provided information on the type of counseling center at which they worked (NG vs. local health office), which was the initial and only compulsory questionnaire item. Thus, the sample size was 145, of which 56 indicated working in a local health center and 89 in an NG counseling center. The number of counselors to whom the invitation was forwarded within each invited center could be obtained from phone calls with 62 centers and was *M* = 2.96 (*SD* = 2.56) in local health offices and *M* = 5.58 (*SD* = 5.07) in NG counseling centers. Five of the selected local health offices did not participate in the survey. Based on this information, the estimated number of counselors invited to participate in the survey was 98 for local health offices and 212 for NG counseling centers. Using these estimates, the response rate was 57.1% for local health offices and 42.0% for NG counseling centers. Of the 145 participants, 77.9% completed the questionnaire in its entirety.

### Demographic Data

Demographic data of the sample are shown in Table 2. The mean age of the participants was 46.0 years (*SD* = 11.7). 76 participants (52.4%) defined themselves as male, 61 (42.1%) as female, and two (1.4%) as gender non-binary. The majority (*n* = 93, 64.1%) indicated that their primary professional qualification was social work; a further 15 (10.3%) indicated that they were physicians, 14 (9.7%) that they were psychologists and four (2.8%) that they were nursing professionals. A large majority of the participants indicated that their counseling center was located in a large city with more than 100,000 inhabitants (*n* = 89, 61.4%) or in a major city with more than 1,000,000 inhabitants (*n* = 43, 29.7%). The vast majority (*n* = 123, 84.8%) indicated that their organization was located in one of the old German states (western Germany) or the city state of Berlin. Statistically significant associations between type of center and demographic data were seen for gender (χ^2^_(df = 2, n = 139)_ = 17,40, *p* < 0.001) and primary professional qualification (χ^2^_(df = 4, *n = 139*)_ = 19,85, *p* = 0.001), see Table 2.

**Table 2:** Demographic data and contextual characteristics of the sample.

**Variable**	**Total sample**		**Type of center**				
			**Local health offices**		**NG counseling centers**		
**N**	145		56		89		
**Age in years (*n* = 139)**							*p* = 0.679^†^
*Mdn* (*IQR*)	48.00	(19.00)	48.00	(17.00)	47.50	(21.75)	
*M* (*SD*)	46.03	(11.67)	46.51	(11.51)	45.75	(11.82)	
*Min*; *Max*	19-67	19-62	23-67								
**Gender (*n*, %)**							*p* < 0.001^§^
Female	61	(42.1%)	34	(60.7%)	27	(30.3%)	
Male	76	(52.4%)	17	(30.4%)	59	(66.3%)	
Non-binary	2	(1.4%)	0	(0%)	2	(2.2%)	
Not specified	6	(4.1%)	5	(8.9%)	1	(1.1%)	
**Professional qualification (*n*, %)**							*p* = 0.001^§^
Social work	93	(64.1%)	37	(66.1%)	56	(62.9%)	
Psychology	14	(9.7%)	2	(3.6%)	12	(13.5%)	
Nursing	4	(2.8%)	1	(1.8%)	3	(3.4%)	
Physician	15	(10.3%)	11	(19.6%)	4	(4.5%)	
Other	13	(9.0%)	0	(0%)	13	(14.6%)	
Not specified	6	(4.1%)	5	(8.9%)	1	(1.1%)	
**Size of the location (*n*, %)**							*p* = 0.138^§^
Major city (>1,000,000)	43	(29.7%)	15	(26.8%)	28	(31.5%)	
Large city (>100,000)	89	(61.4%)	31	(55.4%)	58	(65.2%)	
City (>10,000)	7	(4.8%)	5	(8.9%)	2	(2.2%)	
Small city (≤ 10,000)	1	(0.7%)	1	(1.8%)	0	(0%)	
Not specified	5	(3.4%)	4	(7.1%)	1	(1.1%)	
**Federal state (*n*, %)**							*p* = 0.072^§^
Baden-Wuerttemberg	20	(13.8%)	3	(5.4%)	17	(19.1%)	
Bavaria	22	(15.2%)	8	(14.3%)	14	(15.7%)	
Berlin	15	(10.3%)	7	(12.5%)	8	(9.0%)	
Brandenburg	9	(6.2%)	3	(5.4%)	6	(6.7%)	
Bremen	1	(0.7%)	0	(0%)	1	(1.1%)	
Hamburg	13	(9.0%)	4	(7.1%)	9	(10.1%)	
Hesse	12	(8.3%)	2	(3.6%)	10	(11.2%)	
Mecklenburg-Western Pomerania	2	(1.4%)	2	(3.6%)	0	(0%)	
Lower Saxony	8	(5.5%)	6	(10.7%)	2	(2.2%)	
North Rhine-Westphalia	22	(15.2%)	8	(14.3%)	14	(15.7%)	
Rheinland-Pfalz	0	(0%)	0	(0%)	0	(0%)	
Saarland	4	(2.8%)	3	(5.4%)	1	(1.1%)	
Saxony	0	(0%)	0	(0%)	0	(0%)	
Saxony-Anhalt	1	(0.7%)	0	(0%)	1	(1.1%)	
Schleswig-Holstein	6	(4.1%)	3	(5.4%)	3	(3.4%)	
Thuringia	0	(0%)	0	(0%)	0	(0%)	
Not specified	10	(6.9%)	7	(12.5%)	3	(3.4%)	
**Professional experience in years (n = 138)**							*p* = 0.838^†^
*Mdn* (*IQR*)	11.50	(18.25)	11.00	(17.50)	12.00	(19.75)	
*M* (*SD*)	14.19	(10.38)	14.14	(10.02)	14.23	(10.63)	
*Min*; *Max*	0.5-40		0.5-31		1-40		

*IQR, inter quartile range; M, mean; Max, maximum; Mdn, median; Min, minimum; SD, standard deviation*.

† *From Mann-Whitney U tests of the null hypothesis that the median value of participants from local health offices is equal to that of participants from NG counseling centers*.

§ *From Pearson's Chi squared tests of the null hypothesis that there is no statistically significant difference between the observed and expected frequencies in each category, by type of counseling center*.

### Counseling Sessions and Practice

Table 3 depicts data on the number of counseling sessions and HIV tests reported by the participants. Counselors averaged 36.6 counseling sessions with MSM and trans persons per month (*SD* = 48.2) and 16.0 sessions with MSM and trans persons who met the criteria to be offered PrEP according to the recommendations of the German and Austrian guideline on PrEP (at-risk clients) (*SD* = 22.2). No significant differences were seen with regard to these two variables between NG counseling centers and local health offices. However, counselors from local health offices reported a higher number of HIV tests per month (*Mdn* = 180, *IQR* = 190) than did participants from NG counseling centers (*Mdn* = 47.5, *IQR* = 73.8), *U* = 1103.5, *p* < 0.001. No significant differences between the two types of centers were seen with respect to the absolute or the relative number of positive HIV tests per month.

**Table 3:** Counseling sessions and HIV-tests.

**Variable**	**Total sample**		**Type of center**				
			**Local health office**		**NG counseling center**		
**Number of overall counseling sessions with MSM and trans persons per month (*n* = 126)**							*p* = 0.784^†^
*Mdn* (*IQR*)	20.00	(35.00)	20.00	(40.00)	25.00	(30.00)	
*M* (*SD*)	36.55	(48.23)	39.21	(52.13)	34.96	(46.03)	
*Min*; *Max*	0-330		0-270		0-330		
**Number of sessions with MSM and trans persons who met the criteria to be offered PrEP according to the German and Austrian guideline (at-risk clients) (*n* = 116)**							*p* = 0.780^†^
*Mdn* (*IQR*)	10.00	(10.00)	10.00	(12.50)	10.00	(10.00)	
*M* (*SD*)	15.97	(22.17)	15.38	(18.70)	16.35	(24.23)	
*Min*; *Max*	0-170		0-80		1-170		
**Overall number of HIV tests per month (*n* = 123)**							*p* < 0.001^†^
*Mdn* (*IQR*)	60.00	(175.00)	180.00	(190.00)	47.50	(73.75)	
*M* (*SD*)	112.69	(109.85)	162.81	(116.12)	81.70	(93.87)	
*Min*; *Max*	3-400		3-400		8-350		
**Number of positive HIV test results per month (*n* = 117)**							*p* = 0.311^†^
*Mdn* (*IQR*)	0.00	(1.00)	1.00	(1.00)	0.00	(1.00)	
*M* (*SD*)	0.67	(0.83)	0.78	(0.90)	0.60	(0.78)	
*Min*; *Max*	0-4		0-3		0-4		
**Proportion of positive HIV test results among overall number of HIV tests (*n* = 117)**							*p* = 0.373^†^
*Mdn* (*IQR*)	0.00%	(0.93)	0.33%	(0.65)	0.00%	(1.67)	
*M* (*SD*)	0.74%	(1.49)	0.34%	(0.38)	0.99%	(1.84)	
*Min*; *Max*	0-12.5%		0-1.25%		0-12.5%		

*IQR, inter quartile range; M, mean; Max, maximum; Mdn, median; Min, minimum; SD, standard deviation*.

† *From Mann-Whitney U tests of the null hypothesis that the median value of participants from local health offices is equal to that of participants from NG counseling centers*.

Taking into account the entire sample, the participating counselors indicated on average that in 26.1% of counseling sessions with at-risk clients, the clients themselves had addressed the topic of PrEP (*SD* = 22.0). The proportion of PrEP advice provided proactively by the counselors was indicated to be 52.0% on average (*SD* = 34.2). The proportion of clients addressing the topic of PrEP themselves was larger in NG counseling centers (*Mdn* = 30.0%, *IQR* = 40.0) than in local health offices (*Mdn* = 10.0%, *IQR* = 10.0), *U* = 877.0, *p* < 0.001. Similarly, the proportion of PrEP advice provided proactively by the counselors was larger in NG counseling centers (*Mdn* = 50.0%, *IQR* = 60.0) than in local health offices (*Mdn* = 30.0%, *IQR* = 70.0), *U* = 1082.0, *p* = 0.003. The data are shown in Table 4.

**Table 4:** Counseling practice in counseling sessions with MSM and trans persons who met the criteria to be offered PrEP according to the German and Austrian guideline (at-risk clients).

**Variable**	**Total sample**		**Type of center**				
			**Local health office**		**NG counseling center**		
**Proportion of sessions with ‘at-risk’ MSM and trans persons in which the topic PrEP is addressed by the clients themselves (*n* = 115)**							*p* < 0.001^†^
*Mdn* (*IQR*)	20.00%	(30.00)	10.00%	(10.00)	30.00%	(40.00)	
*M* (*SD*)	26.09%	(21.95)	16.36%	(15.86)	32.11%	(23.11)	
*Min*; *Max*	0-100%		0-80%		0-100%		
**Proportion of sessions with ‘at-risk’ MSM and trans persons in which the counselors themselves proactively address the topic PrEP (*n* = 116)**							*p* = 0.003^†^
*Mdn* (*IQR*)	50.00%	(70.00)	30.00%	(70.00)	50.00%	(60.00)	
*M* (*SD*)	51.98%	(34.24)	41.33%	(36.72)	58.73%	(30.98)	
*Min*; *Max*	0-100%		0-100%		10-100%		

*IQR, inter quartile range; M, mean; Max, maximum; Mdn, median; Min, minimum; SD, standard deviation*.

† *From Mann-Whitney U tests of the null hypothesis that the median value of participants from local health offices is equal to that of participants from NG counseling centers*.

### Self-Assessment of Knowledge and Counseling Competence

For each of the self-assessed dimensions of knowledge and counseling competence, agreement (and hence a positive self-assessment of knowledge and counseling skills regarding PrEP) was more frequent than indifference or disagreement with the respective statements. However, there was a statistically significant association between the type of center and the agreement for each of the aspects assessed (Table 5). For the summative “knowledge score,” Crohnbach's alpha was α = 0.966. The knowledge score was significantly higher for counselors from NG counseling centers (*Mdn* = 18.0, *IQR* = 5.0) than for counselors from local health offices (*Mdn* = 14.0, *IQR* = 4.0), *U* = 679.5, *p* < 0.001.

**Table 5:** Self-assessment of knowledge and counseling competence.

**Variable**	**Total sample**		**Type of center**				
			**Local health office**		**NG counseling center**		
**Global assessment: “I am well-informed about PrEP” (*n*, %), *n* = 113**							*p* < 0.001^§^
Strongly disagree	1	(0.9%)	0	(0.0%)	1	(1.4%)	
Disagree	2	(1.8%)	1	(2.3%)	1	(1.4%)	
Neither agree nor disagree	13	(11.5%)	11	(25.0%)	2	(2.9%)	
Agree	44	(38.9%)	21	(47.7%)	23	(33.3%)	
Strongly agree	53	(46.9%)	11	(25.0%)	42	(60.9%)	
**Indications: “I am able to comprehensively give clients advice on whether it makes sense to take PrEP in their respective case” (*n*, %), *n* = 113**							*p* < 0.001^§^
Strongly disagree	1	(0.9%)	0	(0.0%)	1	(1.4%)	
Disagree	6	(5.3%)	5	(11.6%)	1	(1.4%)	
Neither agree nor disagree	9	(8.0%)	5	(11.6%)	4	(5.7%)	
Agree	38	(33.6%)	22	(51,2%)	16	(22.9%)	
Strongly agree	59	(52.2%)	11	(25.6%)	48	(68.6%)	
**Adverse effects: “I am able to comprehensively give clients advice on the adverse effects of PrEP” (*n*, %), *n* = 113**							*p* < 0.001^§^
Strongly disagree	3	(2.7%)	2	(4.7%)	1	(1.4%)	
Disagree	11	(9.7%)	8	(18.6%)	3	(4.3%)	
Neither agree nor disagree	26	(23.0%)	16	(37.2%)	10	(14.3%)	
Agree	37	(32.7%)	11	(25.6%)	26	(37.1%)	
Strongly agree	36	(31.9%)	6	(14.0%)	30	(42.9%)	
**Modalities of intake: “I am able to comprehensively give clients advice on the possible modalities of intake of PrEP (e.g., continuous vs. on-demand)” (*n*, %), *n* = 113**							*p* < 0.001^§^
Strongly disagree	2	(1.8%)	1	(2.3%)	1	(1.4%)	
Disagree	13	(11.5%)	11	(25.6%)	2	(2.9%)	
Neither agree nor disagree	8	(7.1%)	3	(7.0%)	5	(7.1%)	
Agree	35	(31.0%)	20	(46.5%)	15	(21.4%)	
Strongly agree	55	(48.7%)	8	(18.6%)	47	(67.1%)	
**Investigations: “I am able to comprehensively give clients advice on the medical investigations necessary during the use of PrEP” (*n*, %), *n* = 113**							*p* = 0.002^§^
Strongly disagree	3	(2.7%)	2	(4.7%)	1	(1.4%)	
Disagree	10	(8.8%)	8	(18.6%)	2	(2.9%)	
Neither agree nor disagree	10	(8.8%)	4	(9.3%)	6	(8.6%)	
Agree	37	(32.7%)	18	(41.9%)	19	(27.1%)	
Strongly agree	53	(46.9%)	11	(25.6%)	42	(60.0%)	
**Knowledge score (0-20), *n* = 112**							*p* < 0.001^†^
*Mdn* (*IQR*)	17.00	(6.00)	14.00	(4.00)	18.00	(5.00)	
*M* (*SD*)	15.64	(4.43)	13.30	(4.38)	17.10	(3.82)	
*Min*; *Max*	0-20		4-20		0-20		

*IQR, inter quartile range; M, mean; Max, maximum; Mdn, median; Min, minimum; SD, standard deviation*.

† *From Mann-Whitney U tests of the null hypothesis that the median value of participants from local health offices is equal to that of participants from NG counseling centers*.

§ *From Pearson's Chi squared tests of the null hypothesis that there is no statistically significant difference between the observed and expected frequencies in each category, by type of counseling center*.

### Attitudes Toward PrEP

As with the knowledge and counseling competence aspects presented above, agreement with the dimensions assessed for attitudes toward PrEP was more frequent than indifference or disagreement with the four statements expressing positive attitudes toward PrEP. For the statement expressing a negative attitude, disagreement was more frequent than indifference or agreement. Again, for each of the aspects assessed, significant associations between the type of center and agreement were found (Table 6). For the summative “attitudes score,” Crohnbach's alpha was α = 0.847. The attitudes score was significantly higher for counselors from NG counseling centers (*Mdn* = 18.0, *IQR* = 4.0) than for counselors from local health offices (*Mdn* = 14.0, *IQR* = 6.8), *U* = *638.5, p* < 0.001.

**Table 6:** Attitudes toward PrEP.

**Variable**	**Total sample**		**Type of center**				
			**Local health office**		**NG counseling center**		
**Global assessment: “I think that PrEP is an important element of HIV prevention strategies” (*n*, %), *n* = 114**							*p* < 0.001^§^
Strongly disagree	1	(0.9%)	1	(2.3%)	0	(0.0%)	
Disagree	2	(1.8%)	2	(4.5%)	0	(0.0%)	
Neither agree nor disagree	11	(9.6%)	8	(18.2%)	3	(4.3%)	
Agree	16	(14.0%)	13	(29.5%)	3	(4.3%)	
Strongly agree	84	(73.7%)	20	(45.5%)	64	(91.4%)	
**Reliability: “I think that PrEP is a reliable method to protect oneself from HIV” (*n*, %), *n* = 114**							*p* = 0.003^§^
Strongly disagree	0	(0.0%)	0	(0.0%)	0	(0.0%)	
Disagree	6	(5.3%)	4	(9.1%)	2	(2.9%)	
Neither agree nor disagree	7	(6.1%)	5	(11.4%)	2	(2.9%)	
Agree	33	(28.9%)	18	(40.9%)	15	(21.4%)	
Strongly agree	68	(59.6%)	17	(38.6%)	51	(72.9%)	
**Adverse effects: “I think that PrEP is a method to protect oneself from HIV that has few side effects” (*n*, %), *n* = 114**							*p* = 0.002^§^
Strongly disagree	8	(7.0%)	3	(6.8%)	5	(7.1%)	
Disagree	12	(10.5%)	8	(18.2%)	4	(5.7%)	
Neither agree nor disagree	32	(28.1%)	18	(40.9%)	14	(20.0%)	
Agree	32	(28.1%)	11	(25.0%)	21	(30.0%)	
Strongly agree	30	(26.3%)	4	(9.1%)	26	(37.1%)	
**Availability of better alternatives: “I think that PrEP is unnecessary, because there are better alternatives to protect oneself from HIV” (*n*, %), *n* = 114**							*p* < 0.001^§^
Strongly disagree	67	(58.8%)	14	(31.8%)	53	(75.7%)	
Disagree	30	(26.3%)	18	(40.9%)	12	(17.1%)	
Neither agree nor disagree	11	(9.6%)	7	(15.9%)	4	(5.7%)	
Agree	5	(4.4%)	4	(9.1%)	1	(1.4%)	
Strongly agree	1	(0.9%)	1	(2.3%)	0	(0.0%)	
**Reimbursement of costs: “I think that PrEP should be paid for by the statutory health insurance” (*n*, %), *n* = 114**							*p* < 0.001^§^
Strongly disagree	8	(7.0%)	5	(11.4%)	3	(4.3%)	
Disagree	9	(7.9%)	6	(13.6%)	3	(4.3%)	
Neither agree nor disagree	16	(14.0%)	13	(29.5%)	3	(4.3%)	
Agree	22	(19.3%)	9	(20.5%)	13	(18.6%)	
Strongly agree	59	(51.8%)	11	(25.0%)	48	(68.6%)	
**Attitudes score (0-20) (*n* = 114)**							*p* < 0.001^†^
*Mdn* (*IQR*)	17.50	(5.00)	14.00	(6.75)	18.00	(4.00)	
*M* (*SD*)	15.96	(4.01)	13.57	(4.16)	17.46	(3.10)	
*Min*; *Max*	4-20		4-20		7-20		

*IQR, inter quartile range; M, mean; Max, maximum; Mdn, median; Min, minimum; SD, standard deviation*.

† *From Mann-Whitney U tests of the null hypothesis that the median value of participants from local health offices is equal to that of participants from NG counseling centers*.

§ *From Pearson's Chi squared tests of the null hypothesis that there is no statistically significant difference between the observed and expected frequencies in each category, by type of counseling center*.

### Multiple Linear Regression on the Proportion of Proactive PrEP Advice

A multiple linear regression was modeled to predict the proportion of PrEP advice provided proactively by the counselors to at-risk clients. Applying backward elimination with *p* < 0.2 as a stopping rule for the exclusion of each variable, a significant regression equation was found (*F*_(2,109)_ = 10.50, *p* < 0.001, *n* = 112), with *R*^2^ = 0.162 (Table 7). The only independent predictors that remained in the model were the knowledge and the attitudes score. Participants' predicted proportion of proactive PrEP advice in sessions with at-risk clients was equal to−8.208 + 1.692 (knowledge score) + 2.111 (attitudes score), where knowledge score and attitudes score are coded on scales from 0 to 20 points, with higher scores indicating a more positive self-assessment of knowledge about PrEP and more positive attitudes toward PrEP, respectively, and the proportion of proactive advice on PrEP is coded on a scale from 0 to 100%. The proportion of proactive PrEP advice provided to at-risk clients increased by 1.7% and by 2.1% for each point increase on the knowledge score and on the attitudes score scales, respectively.

**Table 7:** Multiple linear regression to predict the proportion of PrEP advice provided proactively to MSM and trans persons who meet the criteria be offered PrEP according to the German and Austrian guideline (at-risk clients).

**Predictors**	**Coefficient (Robust SE)**		**Beta**	***p***	**VIF**
Constant	−8.208	(11.468)		0.476	
Knowledge score^1^	1.692	(0.842)	0.221	0.047	1.26
Attitudes score^2^	2.111	(0.910)	0.250	0.022	1.26

*SE, standard error; VIF, variance inflation factor*.

1 *Scale from 0 to 20 points, with higher scores indicating a more positive self-assessment of knowledge about PrEP and counseling competence*.

2 *Scale from 0 to 20 points, with higher scores indicating a more positive attitudes toward PrEP*.

### Guidelines, Training and Educational Material

Slightly fewer than half of the participants (48.7%, *n* = 55) indicated that their respective organization had in-house PrEP guidelines or standard operating procedures, but a large majority indicated that training on PrEP advice had been offered to them (86.0%, *n* = 98). Fewer than half of the participants indicated that they wished to receive further training on PrEP counseling (44.6%, *n* = 50). Counselors from NG counseling centers indicated having been offered training on PrEP advice more frequently (90.0%, *n* = 63) than counselors from local health offices (79.5%, *n* = 35), but this difference was not statistically significant (χ^2^_(df = 1, *n* = 114)_ = 2,447, *p* = 0.118). Regarding the availability of in-house guidelines and the wish for further training on PrEP no significant differences by type of center were seen, likewise. Asked which of the listed information materials or trainings would improve their counseling practice, decision aids for the clients that present information on PrEP in client-friendly language and in different languages were chosen most frequently (both 78.8%, *n* = 89), followed by a clinical practice guideline that provides a good overview of indications, contraindications and necessary investigations (74.3%, *n* = 84). Less frequently mentioned materials or training were: an app- or SMS-based reminder for PrEP users to promote adherence (58.4%, *n* = 66), information and training for counselors on the management of PrEP (45.1%, *n* = 51), information and training for counselors on the identification of PrEP candidates (38.1%, *n* = 43), and information and training for counselors on talking with clients about sexuality (28.3%, *n* = 32).

Asked to rate the relevance of barriers for potential users to initiate PrEP as perceived in their personal counseling practice, participants pointed to worries about getting infected with other sexually transmitted infections (*M* = 5.56, *SD* = 2.73), the monthly cost of the PrEP medication (*M* = 5.33, *SD* = 2.61), and a lack of information about PrEP in the native language of the client (*M* = 5.10, *SD* = 3.33). Further results on perceived barriers to initiate PrEP are shown in Table 8.

**Table 8:** Relevance of barriers to PrEP use.

	***n***	**M (SD)**		**Min-Max**
Worries about getting infected with other STIs	111	5.56	(2.73)	0-10
The monthly costs for the PrEP medication	109	5.33	(2.61)	0-10
Lack of information about PrEP in the native language of the client	110	5.10	(3.33)	0-10
The costs for the laboratory investigations	109	4.80	(3.00)	0-10
Worries about mild or temporary side effects	109	4.64	(2.43)	0-10
Time required for regular visits to the doctor	111	4.26	(2.81)	0-10
Worries about severe or permanent side effects	111	4.21	(2.59)	0-10
Lack of information about PrEP in client-friendly language	110	4.17	(2.88)	0-10
Difficulties finding a doctor who prescribes PrEP	112	4.13	(3.64)	0-10
Assessment of the own risk of getting infected with HIV as too low to take PrEP	110	4.08	(2.70)	0-10
Worries about stigmatization in the peer group	107	3.33	(2.67)	0-10
Cultural barriers	110	2.79	(2.51)	0-10

*M, mean; Max, maximum; Min, minimum; SD, standard deviation*.

## Discussion

This is the first survey to assess knowledge, attitudes and counseling practice regarding PrEP among counselors from HIV and STI testing and counseling centers in Germany. Given that targeted counseling of persons at increased risk of acquiring HIV can help them take an informed decision about their personal HIV prevention strategy, counseling centers can play a key role in improving the implementation of PrEP. Providing persons at risk of HIV infection with reliable information on PrEP is an essential prerequisite for improving PrEP implementation in Germany. For this study, we focused on MSM and trans persons who met the criteria to be offered PrEP according to the guideline currently applicable in Germany, and the proportion of PrEP advice proactively provided to this group was one of the key outcomes evaluated within our study.

Regardless of whether they were employed in NG counseling centers or local health offices, participants in the survey indicated that they indeed had counseling sessions with these “at-risk clients” and that they proactively provided PrEP advice in sessions with this group of clients, albeit to varying degrees. The majority of the participating counselors had a positive self-assessment of their knowledge and counseling skills as well as positive attitudes toward PrEP. However, significant differences were found between counselors from NG counseling centers and local health offices: the self-assessment indicated that the former had greater knowledge and counseling skills and more positive attitudes toward PrEP. Furthermore, the proportion of PrEP advice provided proactively in sessions with at-risk clients was larger among counselors from NG counseling centers than among counselors from local health offices.

The differences found between NG counseling centers and local health offices may be attributable to a different basic orientation and organizational policy: whereas NG counseling centers arose from community-based self-help organizations, the local health offices have long focused on advice on HIV and STIs for the overall population and selected risk groups such as sex workers. Whereas the majority of clients in NG counseling centers are MSM (36), this client group only constitutes a minority of the clients in local health offices (37). In the multiple linear regression, however, knowledge of and attitudes toward PrEP remained the only independent predictive factors for the proportion of PrEP advice provided proactively in sessions with at-risk clients. This implies that the differences between the two types of centers are mainly explained by different knowledge and counseling skills and attitudes toward PrEP on the side of the individual counselors working in the respective centers. This finding points at the importance of training for the counselors and of supplying material that facilitates counseling on PrEP.

Overall, the counselors participating in the survey indicated that they proactively provided PrEP advice in a mean of 52.0% of sessions with at-risk clients, and it must therefore be assumed that the implementation of the current German and Austrian PrEP guideline has been incomplete so far. This assumption is supported by the fact that, despite of their existence, almost three quarters of the participants indicated that a guideline with a clear presentation of indications, contraindications and necessary laboratory investigations would help to improve PrEP consultations. The wording of the indication for recommending PrEP to MSM and trans persons (“MSM or trans persons who report having had anal sex without condom within the past 3-6 months and/or probably having anal sex without condom in the next months, or who had an STI in the last 12 months, respectively,” English translation by the authors of the present paper) in the German and Austrian guideline (19) is ambiguous due to the use of unclear operators (“and/or,” “respectively”) and imprecisely defined time periods (“3-6 months”, “next months”). This may be a factor that limits the implementation of the guideline recommendations. The survey revealed that fewer than half of the centers had in-house guidelines or standard operating procedures for PrEP counseling. No information was collected on the content of these in-house guidelines, and it remains unclear whether they contain indications for PrEP advice that deviate from the German and Austrian guidelines. For the purpose of this study, the recommendations of the German and Austrian guideline on HIV pre-exposure prophylaxis were used to define at-risk clients. Whereas one indication to offer PrEP to MSM and trans persons according to the German and Austrian Guideline is a history of an STI in the past 12 months (19), the CDC guidelines on PrEP, for instance, restrict this aspect to the past six months and exclusively to bacterial STIs (18). A narrower definition of the indication to recommend PrEP to MSM and trans people might have led to a higher proportion of proactive PrEP advice in counseling sessions with these clients. It must also be taken into account that the German and Austrian guideline on PrEP has only been available since June 2018, and thus for approximately four months before the data collection for this survey began. This relatively short period of time is probably the most important reason for the incomplete implementation of the guideline recommendations in current counseling practice and for the limited awareness of the guideline found in this survey.

Nonetheless, the incomplete implementation of the current guideline recommendations and the limited awareness of their existence indicate that there is a need and potential for improving and harmonizing counseling on PrEP in counseling centers, particularly when targeting at-risk populations. Bearing this in mind, it is interesting that the counselors who took part in the survey selected mainly client-directed tools as resources that would help to improve PrEP counseling. Among the most frequently selected tools were (1) decision aids for clients that provide information about PrEP in client-friendly or (2) in the client's first language, and (3) an app- or SMS-based reminder system for PrEP users to promote their adherence. In contrast, information or training for counselors was less frequently selected as being helpful for their counseling practice. In line with these results, fewer than half of the participants indicated that they would like to receive training or courses on PrEP counseling. This must be taken into account when deciding on measures to improve targeted counseling on PrEP among counselors in sexual health services.

The focus on client-directed information material and tools when selecting resources that would improve counseling on PrEP reflects that lack of information on the side of potential PrEP users is perceived as one of the most important barriers to initiating PrEP. This barrier can be addressed through the availability of easily understandable information material for clients and especially populations at risk of acquiring HIV. Concerns about sexually transmitted infections, the cost of PrEP medication and follow-up examinations, the lack of information about PrEP in the clients' first languages and worries about mild or temporary side effects were among the barriers for potential PrEP users rated as particularly relevant by the counselors who participated in this survey. This corresponds well with the barriers to taking PrEP found in the Berlin survey among MSM (28). However, aspects such as the costs of PrEP medication and corresponding accompanying examinations as barriers to initiating PrEP are structural barriers. With a law passed in July 2019, the cost of PrEP and necessary laboratory investigations has been covered by public health insurance in Germany since September 2019, which renders this barrier obsolete. Stigmatization of PrEP users by their peers or in their social environments was rated by the counselors as the least relevant barrier, although the aspect of stigmatization was repeatedly mentioned in the free text fields and also in the international literature (38).

### Limitations

These insights into PrEP-related knowledge, counseling skills, attitudes, and counseling practice among counselors working in HIV testing and counseling centers can be used to identify and develop strategies for improving PrEP implementation in at-risk populations. However, there is a number of important limitations to consider when interpreting the results:

Firstly, the questionnaire used in this study was not formally validated before it was used as a survey instrument. PrEP-related knowledge and counseling skills were self-assessed by the participants. We did not present a score that assessed specifically defined levels of competence or skills. It is therefore unclear whether the respective score validly represents the actual knowledge and counseling skills. A systematic review showed that there may be relevant discrepancies between self-assessed knowledge and actual knowledge (39). In addition, no empirical data are available on the question of whether the actual quality of counseling on PrEP is determined primarily by the knowledge of the counselors. However, the fact that there was a significant association between the knowledge score and the attitudes score on one hand, and the proportion of proactive PrEP advice in sessions with at-risk clients on the other, indicate that the knowledge and attitudes scores may be a valid representation of the respective concepts. This is also supported by the good internal consistency of the scores.

Secondly, for pragmatic reasons, the risk groups of “MSM” and “trans persons” were grouped together in the survey. As a result, information may have been lost or recorded inaccurately. The assessment of the counseling practice could lead to different findings if the questions had specifically related to the respective populations separately. Especially with regard to the efficacy and safety of PrEP, far more data are available for MSM than for trans persons (9–12, 14–17, 39). At the same time, for trans persons, other access barriers to health care may be relevant than for MSM—for example, for trans persons, finding a competent physician was described as a particularly relevant barrier to accessing PrEP (40). Furthermore, the sexual orientation of the counselors was not assessed in the survey, although this may have a relevant impact on the PrEP counseling practice and explain differences in this regard between counseling centers and health authorities as an additional variable. We also did not include the primary professional qualification of the counselors in our multiple regression model, as this was not one of the variables that we had chosen a priori, which were limited in number to avoid overfitting.

A third limitation, which pertains to the validity of our findings may be the presence of selection bias. Counselors with little knowledge of or negative attitudes toward PrEP may have been less likely to participate in the survey than counselors with more positive attitudes and/or better knowledge. The extent of such a bias cannot be quantified. In this context, it is worth pointing out that the response rates of 42 and 57% for NG counseling centers and local health offices, respectively, were comparatively high for a survey of this nature. For example, surveys on PrEP among physicians in the USA and the Netherlands had response rates of 23.5 and 39%, respectively (30, 32). While high response rates cannot guarantee unbiased estimates, they do provide less opportunity for selection bias to occur. However, the sample size of the present study is relatively small, also limiting the generalisability of our findings. A further limitation of the representativeness is that only few counseling centers from the new German states (former East Germany) took part in the survey. It must be taken into account that access to HIV tests and advice in rural regions and particularly in the new German states is often only supplied by the local health authorities and only in a small number of NG counseling centers. In contrast, large cities such as Berlin and Hamburg have a higher number of NG counseling centers (34). The regional distribution of the participants in the survey therefore reflects the current situation with respect to sexual health services.

### Conclusions

The results of this first survey assessing PrEP-related knowledge, attitudes, and counseling practice among counselors from HIV and STI testing and counseling centers in Germany should be interpreted as baseline data shortly after publication of the German and Austrian guidelines on PrEP. The survey revealed that PrEP counseling in these centers is currently heterogeneous and that the knowledge of and attitudes toward PrEP vary substantially among counselors. In particular, substantial differences were found between counselors from NG counseling centers and the local health offices. Due to the rapid developments in the field of PrEP services in Germany, re-evaluating counseling practice after the guideline recommendations have been available for a longer period and some time after the inclusion of PrEP in the benefits catalog of the public health insurance will probably yield useful findings. In the meantime, concepts that increase the awareness of the guideline recommendations among counselors in the HIV and STI counseling and testing centers in Germany should be developed and implemented. For the comprehensive and successful implementation of HIV prevention strategies with the goal of empowering at-risk populations to take informed decisions, targeted and proactive PrEP advice is a key element. In this regard, there is room for improvement, and both NG counseling centers and the public health authorities should undertake measures to optimize their counselors' knowledge and counseling skills. Bearing in mind that the desire for further training on PrEP counseling was expressed by fewer than half of the counselors who took part in the survey, these measures may focus on decreasing the barriers identified for potential PrEP users, for example by developing and testing resources and tools such as decision-aids for potential PrEP users in client-friendly language and in different languages. Both potential PrEP users and counselors should be included in this process in order to ensure good acceptance and implementation of the tools that are developed.

## Data Availability Statement

All datasets generated for this study are included in the article/Supplementary Material.

## Ethics Statement

The study protocol was reviewed and approved by Ethikkommission der Charité-Universitätsmedizin Berlin. The participants provided their informed consent to participate in this study.

## Author Contributions

FK developed the study design and drafted the questionnaire, which was discussed and finalized by FK, RW, MG, MS, and AN. FK led the data collection. FK, MG, and RW conducted the statistical analyses and supported interpretation of results. FK wrote the first draft of the manuscript. All authors provided considerable editing, revisions and content review of the initial manuscript draft, and approved the final draft of the manuscript.

## Conflict of Interest

The authors declare that the research was conducted in the absence of any commercial or financial relationships that could be construed as a potential conflict of interest.

## Supplementary Material

Data Sheet S1 Survey questionnaire (English translation, please note that the questionnaire was only available in German language and the present translation has been undertaken for the publication only).

Data Sheet S2 Survey questionnaire (original German version).

Table S1 Minimal underlying data set (Please note that gender, age and years of professional experience of the respondents as well as federal state of the counseling center and qualitative data have been removed to ensure the anonymity of the respondents).
