# Supplementary material for: HIV Pre-Exposure Prophylaxis (PrEP) Counseling in Germany: Knowledge, Attitudes and Practice in Non-governmental and in Public HIV and STI Testing and Counseling Centers
Source: Front Public Health. 2020 Jul 14;8:298. doi: 10.3389/fpubh.2020.00298 (PMC7372106; doi:10.3389/fpubh.2020.00298)
Supplement: Data Sheet S1 — Survey questionnaire (English translation, please note that the questionnaire was only available in German language and the present translation has been undertaken for the publication only). [file Data_Sheet_1.PDF]

Supporting material 1: Survey questionnaire (English translation*)

Kutscha F, Gaskins M, Sammons M, Nast A, Werner RN: HIV pre-exposure prophylaxis (PrEP) counselling in Germany: Knowledge, attitudes and practice in nongovernmental and in public HIV and STI testing and counselling centres

* Please note that the questionnaire was only available in German language and the present translation has been undertaken for the publication only.

--

Attitudes and counselling practice regarding HIV pre-exposure prophylaxis (PrEP) among counsellors working in HIV and STI counselling and testing centres in Germany

We would like to give you the opportunity to participate in an anonymous survey of the attitudes and counselling practice regarding HIV pre-exposure prophylaxis (PrEP) among counsellors working in HIV and STI counselling and testing centres in Germany.

The aim of this study is to assess what counsellors think about PrEP and what problems arise when counselling clients. The study aims at identifying difficulties, potential for improvement, and thus, at contributing to the prevention of HIV infections in gay men and other (trans*) men who have sex with men.

The survey is anonymous and takes approximately 10 minutes to complete.

Participation in the survey is voluntary. You can cancel your participation at any time without giving a reason.

Please only take part in this survey if you work as a counsellor in the area of HIV and STI testing and counselling. Please complete the survey only once.

What is the survey about?

HIV pre-exposure prophylaxis (PrEP) is a form of prevention of infections with HIV that has been approved in Germany in August 2016. The continuous or on-demand intake of the combination of tenofovir disoproxil and emtricitabine provides protection against HIV infection during sex even without using condoms.

The high efficacy and safety of PrEP has been shown in various randomized controlled studies and cohort studies. The implementation of PrEP has already led to a decrease in the incidence of new HIV infections among men who have sex with men in some major cities (e.g. London, San Francisco). PrEP also plays an increasing role in Germany. Due to the expected inclusion of PrEP in the benefits catalogue of the statutory health insurance, an increasing number of consultations on the topic is expected.

Data protection

We guarantee your anonymity. No information is collected that can be used to identify you personally.

The data of the completed anonymous questionnaire are transmitted in encrypted form and statistically evaluated. A report of the survey is expected to be issued by mid-2019.

No data will be published that allow conclusions about the individual participants or their employers.

The report is to be published in a scientific journal. Additional reports of the survey results may be published at medical congresses, in magazines, and / or on the websites of organizations.

*** If you want to participate, please confirm:**
O I have read the study information and would like to take part in this survey.

Some questions about you first...

What is your gender?
O Female
O Male
O Gender non-binary

How old are you?
______ years

What is you primary professional qualification?
o Social work
o Psychology
o Nursing
o Physician
o Other: _________________

How many years of professional experience do you have in sexual health counselling?
approx. ______ years

... and some questions about the counselling centre you work at:

*** What type of HIV and STI counselling centre do you work at?**
O Local health office (for example public health department, local health authorities)
O NGO counselling centre (for example AIDS Hilfe)
O Other: __________________

How many counsellors work in your facility?

______

Please indicate the state of your counselling centre. *(drop down menu)*
– Baden-Wuerttemberg – Bavaria – Berlin – Brandenburg – Bremen – Hamburg – Hesse – Mecklenburg-Western Pomerania – Lower Saxony – North Rhine-Westphalia – Rheinland-Pfalz – Saarland – Saxony – Saxony-Anhalt – Schleswig-Holstein – Thuringia

Where is your counselling centre located?
O In a major city (> 1,000,000 inhabitants)
O In a large city (> 100,000 inhabitants)
O In a city with more than 10,000 and less than 100,000 inhabitants
O In a city with less than 10,000 inhabitants or in a rural region

---

Some questions about your day-to-day work...

Please estimate:
How many HIV tests per month are performed in your counselling centre on average?
approx. _______

Please estimate:
How many persons per month are tested positive for HIV in your counselling centre on average?
approx. _______

Please estimate:
How many counselling contacts with men who have sex with men (MSM) and trans people per month do you personally have on average?
approx. _______

…and about your counselling practice

The German-Austrian guidelines on HIV pre-exposure prophylaxis* recommend PrEP for all HIV-negative MSM or transgender people who meet at least one of the following criteria:

- History of anal sex without a condom in the past 3-6 months
- Indication of likely having anal sex without a condom in the next months
- History of a sexually transmitted infection (STI) in the past 12 months

* German-Austrian guidelines on HIV pre-exposure prophylaxis, Deutsch-Österreichische Leitlinien zur HIV-Präexpositionsprophylaxe, AWMF-Register-Nr.: 055-008, https://daignet.de/site-content/hiv-therapie/leitlinien-1

The following three questions relate to your personal counselling contacts with clients who meet these criteria.

Please estimate:
How many counselling contacts with clients who meet the above criteria do you personally have on average in a month?
approx. _____

Please estimate:
In how many of these counseling contacts <u>do clients themselves</u> address the desire to take PrEP? *(drop down menu)*
- 0% - 10% - 20% - 30% - 40% - 50% - 60% - 70% - 80% - 90% - 100%

Please estimate:
In how many of these counseling contacts <u>do you as a counsellor</u> address that PrEP could be a suitable measure to protect against an infection with HIV? *(drop down menu)*
- 0% - 10% - 20% - 30% - 40% - 50% - 60% - 70% - 80% - 90% - 100%

<u>Some questions about your knowledge and attitudes towards PrEP</u>

Were counsellors working in your counselling centre offered internal or external training on the subject of PrEP and counselling on PrEP?
O yes
O no

Would you like to receive training or courses on counselling clients about PrEP?
O yes
O no

Do you agree or disagree with the following statements? *(items presented in randomised order)*

	Strongly disagree	*Disagree*	*Neither agree nor disagree*	*Agree*	*Strongly agree*
"I am well-informed about PrEP"	O	O	O	O	O
"I am able to comprehensively give clients advice on whether it makes sense to take PrEP in their respective case"	O	O	O	O	O
"I am able to comprehensively give clients	O	O	O	O	O

advice on the adverse effects of PrEP"					
"I am able to comprehensively give clients advice on the possible modalities of intake of PrEP (eg, continuous vs. on-demand)"	O	O	O	O	O
"I am able to comprehensively give clients advice on the medical investigations necessary during the use of PrEP"	O	O	O	O	O

Are internal guidelines or procedural instructions for PrEP counselling available at your counselling centre?
O yes
O no

Do you agree or disagree with the following statements? *(items presented in randomised order)*

	Strongly disagree	*Disagree*	*Neither agree nor disagree*	*Agree*	*Strongly agree*
"I think that PrEP is an important element of HIV prevention strategies"	O	O	O	O	O
"I think that PrEP is a reliable method to protect oneself from HIV"	O	O	O	O	O
"I think that PrEP is a method to protect oneself from HIV that has few side effects"	O	O	O	O	O
"I think that PrEP is unnecessary, because there are better alternatives to protect oneself from HIV"	O	O	O	O	O
"I think that PrEP should be paid for by the statutory health insurance"	O	O	O	O	O

<u>Almost finished. Now some questions about the perspective of your clients ...</u>

How relevant are the following problems for potential PrEP users according to your personal counselling experience? *(items presented in randomised order)*

	No relevance – 0	*1*	*2*	*3*	*4*	*5*	*6*	*7*	*8*	*9*	*Highest relevance – 10*
Worries about getting infected with other STIs	O	O	O	O	O	O	O	O	O	O	O
The monthly costs for the PrEP medication	O	O	O	O	O	O	O	O	O	O	O
Lack of information about PrEP in the native language of the client	O	O	O	O	O	O	O	O	O	O	O
The costs for the laboratory investigations	O	O	O	O	O	O	O	O	O	O	O
Worries about mild	O	O	O	O	O	O	O	O	O	O	O

or temporary side effects											
Time required for regular visits to the doctor	O	O	O	O	O	O	O	O	O	O	O
Worries about severe or permanent side effects	O	O	O	O	O	O	O	O	O	O	O
Lack of information about PrEP in client-friendly language	O	O	O	O	O	O	O	O	O	O	O
Difficulties finding a doctor who prescribes PrEP	O	O	O	O	O	O	O	O	O	O	O
Assessment of the own risk of getting infected with HIV as too low to take PrEP	O	O	O	O	O	O	O	O	O	O	O
Worries about stigmatization in the peer group	O	O	O	O	O	O	O	O	O	O	O
Cultural barriers	O	O	O	O	O	O	O	O	O	O	O

Are there any other relevant problems for potential PrEP users that occur in your personal counseling practice?
Please specify these here... ____________________________________

Two final questions:

Which of the following information or training materials would improve counselling on PrEP or make it more practical? *(Multiple answers possible)*

O A clinical practice guideline that provides a good overview of indications, contraindications and necessary laboratory investigations
O A decision-aid for clients that provides information on PrEP in client-friendly language
O A decision-aid for clients that provides information on PrEP in different languages
O Information and training for counsellors on the management of PrEP (e.g. required examinations etc.)
O Information and training for counsellors on the identification of PrEP candidates
O Information or training on the subject of “Talking with clients about sexuality”
O An app- or SMS-based reminder for PrEP users to promote adherence
O Other: ____________

Are there other aspects or problems that concern you in your counselling work on PrEP? Do you have any other suggestions on how to improve counselling for people who may benefit from PrEP?

Please specify these here ... ___________________

Thank you for participating in the survey!
