# Supplementary material for: HIV Pre-Exposure Prophylaxis (PrEP) Counseling in Germany: Knowledge, Attitudes and Practice in Non-governmental and in Public HIV and STI Testing and Counseling Centers
Source: Front Public Health. 2020 Jul 14;8:298. doi: 10.3389/fpubh.2020.00298 (PMC7372106; doi:10.3389/fpubh.2020.00298)
Supplement: Data Sheet S2 — Survey questionnaire (original German version). [file Data_Sheet_2.PDF]

Supporting material 2: Survey questionnaire (German version)

Kutscha F, Gaskins M, Sammons M, Nast A, Werner RN: HIV pre-exposure prophylaxis (PrEP) counselling in Germany: Knowledge, attitudes and practice in nongovernmental and in public HIV and STI testing and counselling centres

--

Einstellungen und Beratungspraxis zur HIV-Präexpositionsprophylaxe (PrEP) unter Berater*innen in Deutschland

Wir möchten Sie einladen, an einer anonymen Fragenbogen-Studie zum Thema "Einstellungen und Beratungspraxis zur HIV-Präexpositionsprophylaxe (PrEP) unter Berater*innen in Deutschland" teilzunehmen.

Ziel der Studie ist es, zu ermitteln, was Berater*innen zur PrEP denken und welche Probleme sich in der Beratung von Klient*innen ergeben. Die Studie soll Schwierigkeiten und Verbesserungspotenziale aufzeigen und so einen Beitrag zur Prävention von HIV-Infektionen bei schwulen Männern und anderen (trans*) Männern, die Sex mit Männern haben, leisten.

Die Studie besteht aus einem anonymen Online-Fragebogen – es dauert etwa 10 Minuten, den Fragebogen auszufüllen.

Die Teilnahme ist freiwillig. Sie können Ihre Teilnahme jederzeit ohne Angabe von Gründen beenden.

Bitte nehmen Sie an dieser Umfrage nur teil, wenn Sie als Berater*in im Bereich der HIV- und STI-Testung und -Beratung tätig sind. Bitte füllen Sie diesen Fragebogen nur einmal aus.

Worum geht es?

Die HIV-Präexpositionsprophylaxe (PrEP) ist eine seit August 2016 in Deutschland zugelassene Form der Prävention von Infektionen mit HIV. Durch die vorbeugende kontinuierliche oder Risikokontakt-bezogene Einnahme der Kombination zweier Wirkstoffe (Tenofovir disoproxil und Emtricitabin), besteht ein Schutz vor HIV-Infektion bei Sex auch ohne die Verwendung von Kondomen.

Die hohe Wirksamkeit und Sicherheit der PrEP konnte in verschiedenen randomisierten Studien und Kohortenstudien gezeigt werden. Die zunehmende Verbreitung der PrEP hat in einzelnen Großstädten (z.B. London, San Francisco) bereits zu einer Verringerung der Rate an HIV-Neuinfektionen unter Männern, die Sex mit Männern haben, geführt.

Auch in Deutschland spielt die PrEP als HIV-Präventionsstrategie eine zunehmende Rolle. Durch die mögliche Übernahme der Kosten für die Versorgung mit der PrEP durch die gesetzlichen Krankenkassen ist von einer steigenden Anzahl an Beratungen zum Thema auszugehen.

Datenschutz

Wir garantieren Ihnen Anonymität. Es werden keine Informationen über Sie erhoben, durch die Sie persönlich identifiziert werden können.

Die Daten des ausgefüllten anonymen Fragebogens werden verschlüsselt übermittelt und statistisch ausgewertet. Ein Bericht der Umfrage wird voraussichtlich bis Mitte 2019 erstellt.

Es werden keine Daten veröffentlicht, die einen Rückschluss auf die einzelnen Teilnehmer*innen oder ihre Arbeitgeber ermöglichen.

Der Bericht soll in einer wissenschaftlichen Zeitschrift publiziert werden. Möglicherweise werden weitere Berichte von den Umfrageergebnissen auf medizinischen Kongressen, in Magazinen und/oder auf den Internetseiten gemeinnütziger Einrichtungen veröffentlicht.

*** Bitte kreuzen Sie an:**
O Ich habe die Studieninformation gelesen und möchte an dieser Umfrage teilnehmen.

Zunächst einige Fragen zu Ihnen…

Welchem Geschlecht ordnen Sie sich zu?
O Weiblich
O Männlich
O Divers

Wie alt sind Sie?
______ Jahre

Welcher Berufsgruppe gehören Sie an?
O Soziale Arbeit/Sozialpädagogik

O Psychologie
O Pflege
O Humanmedizin
O Andere: _________________

Über wie viele Jahre Berufserfahrung verfügen Sie im Bereich der Beratung in Fragen sexueller Gesundheit?
ca. ______ Jahre

… und zu Ihrer Einrichtung

*** In welcher Trägerschaft befindet sich Ihre Einrichtung?**
O Öffentliche Einrichtung (z.B. Gesundheitsamt, kommunaler Träger)
O Freie Trägerschaft (z.B. AIDS-Hilfen, eingetragener Verein)
O Andere: __________________

Wie viele Mitarbeitende mit Beratungsfunktion arbeiten in Ihrer Einrichtung?
______

Bitte geben Sie das Bundesland Ihres Arbeitsortes an. *(drop down Menü)*
– Baden-Württemberg – Bayern – Berlin – Brandenburg – Bremen – Hamburg – Hessen - Mecklenburg-Vorpommern – Niedersachsen - Nordrhein-Westphalen - Rheinland-Pfalz – Saarland – Sachsen - Sachsen-Anhalt - Schleswig-Holstein – Thüringen

Wo arbeiten Sie?
O In einer Metropole (>1.000.000 Einwohner)
O In einer Großstadt (>100.000 Einwohner)
O In einer Stadt mit mehr als 10.000 und weniger als 100.000 Einwohnern
O In einer Stadt mit weniger als 10.000 Einwohnern oder einer ländlichen Region

---

Einige Fragen zu Ihrem Beratungsalltag...

Bitte schätzen Sie:
Wie viele HIV-Tests werden in Ihrer Einrichtung durchschnittlich in einem Monat durchgeführt?
ca. _______

Bitte schätzen Sie:
Wie viele Personen werden in Ihrer Einrichtung durchschnittlich in einem Monat positiv auf HIV getestet?
ca. _______

Bitte schätzen Sie:
Wie viele Beratungskontakte mit Männern, die Sex mit Männern haben (MSM) und Transpersonen haben Sie selbst durchschnittlich in einem Monat?
ca. _______

... und zu Ihrer Berufspraxis

Die Deutsch-Österreichischen Leitlinien zur HIV-Präexpositionsprophylaxe* empfehlen die PrEP unter anderem für alle <u>HIV-negativen MSM oder Transgender-Personen</u>, auf die mindestens eines der folgenden Kriterien zutrifft:

- Angabe von analem Sex ohne Kondom innerhalb der letzten 3-6 Monate
- Angabe, voraussichtlich in den nächsten Monaten analen Sex ohne Kondom zu haben
- Diagnose einer sexuell übertragbaren Infektion (STI) in den letzten 12 Monaten

* Deutsch-Österreichische Leitlinien zur HIV-Präexpositionsprophylaxe, AWMF-Register-Nr.: 055-008, https://daignet.de/site-content/hiv-therapie/leitlinien-1

Die folgenden drei Fragen beziehen sich auf Ihre persönlichen Beratungskontakte mit Menschen, auf die diese Kriterien zutreffen.

Bitte schätzen Sie:
Wie viele Beratungskontakte mit Klient*innen, auf die die oben genannten Kriterien zutreffen, haben Sie durchschnittlich in einem Monat?
ca. _____

Bitte schätzen Sie:
In wie vielen dieser Beratungskontakte sprechen <u>die Klient*innen eigenständig</u> den Wunsch an, PrEP einzunehmen? (drop down Menü)
- 0% - 10% - 20% - 30% - 40% - 50% - 60% - 70% - 80% - 90% - 100%

Bitte schätzen Sie:
In wie vielen dieser Beratungskontakte <u>sprechen Sie als Berater*in</u> an, dass PrEP eine geeignete Maßnahme sein könnte, sich vor HIV zu schützen? (drop down Menü)
- - 0% - 10% - 20% - 30% - 40% - 50% - 60% - 70% - 80% - 90% - 100%

<u>Einige Fragen zu Ihren Kenntnissen und Einstellungen zur PrEP</u>

Wurden den Mitarbeitenden in Ihrer Einrichtung interne oder externe Fortbildungen oder Schulungsveranstaltungen zum Thema PrEP angeboten?
- o Ja
- o Nein

Wünschen Sie sich mehr Fortbildungen oder Schulungen zum Thema PrEP?
- o Ja
- o Nein

Wie sehr stimmen Sie den folgenden Aussagen zu? *(Items in randomisierter Reihenfolge präsentiert)*

	Stimme gar nicht zu	*Stimme eher nicht zu*	*Teils-teils*	*Stimme eher zu*	*Stimme voll zu*
„Ich kenne mich gut mit PrEP aus“	O	O	O	O	O
„Ich kann Klient*innen umfassend dazu beraten, ob in ihrem	O	O	O	O	O

jeweiligen Fall die Einnahme von PrEP sinnvoll ist“					
„Ich kann Klient*innen zu den möglichen Nebenwirkungen der PrEP umfassend beraten“	O	O	O	O	O
„Ich kann Klient*innen zu den möglichen Einnahmemodalitäten der PrEP (z.B. kontinuierlich oder Risikokontakt-bezogen) umfassend beraten“	O	O	O	O	O
„Ich kann Klient*innen zu den erforderlichen medizinischen Begleituntersuchungen zur PrEP umfassend beraten“	O	O	O	O	O

Gibt es in Ihrer Einrichtung interne Leitlinien oder Verfahrensanweisungen für Beratungen zum Thema PrEP?

- o Ja
- o Nein

Wie sehr stimmen Sie den folgenden Aussagen zu? *(Items in randomisierter Reihenfolge präsentiert)*

	Stimme gar nicht zu	*Stimme eher nicht zu*	*Teils-teils*	*Stimme eher zu*	*Stimme voll zu*
„Ich denke, die PrEP ist ein wichtiger Bestandteil von Präventionsstrategien gegen HIV“	O	O	O	O	O
„Ich denke, die PrEP ist eine verlässliche Methode, sich vor HIV zu schützen"	O	O	O	O	O
„Ich denke, die PrEP ist eine nebenwirkungsarme Methode, sich vor HIV zu schützen“	O	O	O	O	O
„Ich denke, PrEP ist unnötig, denn es gibt bessere Alternativen, um sich vor HIV zu schützen"	O	O	O	O	O
„Ich finde, die Versorgung mit PrEP sollte von der	O	O	O	O	O

gesetzlichen Krankenkasse bezahlt werden“					

Fast fertig. Jetzt noch zur Perspektive Ihrer Klient*innen...

Wie relevant sind in Ihrer persönlichen Beratungspraxis und -Erfahrung die folgenden Probleme für potenzielle PrEP-Nutzer*innen?

	Keine Relevanz – 0	*1*	*2*	*3*	*4*	*5*	*6*	*7*	*8*	*9*	*Höchste Relevanz – 10*
Die Klient*innen haben Sorgen vor leichten/vorübergehenden Nebenwirkungen	O	O	O	O	O	O	O	O	O	O	O
Die Klient*innen haben Sorgen vor schweren/bleibenden Nebenwirkungen	O	O	O	O	O	O	O	O	O	O	O
Die Klient*innen haben Sorgen, sich mit anderen sexuell übertragbaren Erkrankungen zu infizieren	O	O	O	O	O	O	O	O	O	O	O
Die monatlichen Kosten für die PrEP-Medikamente sind für die Klient*innen zu hoch	O	O	O	O	O	O	O	O	O	O	O
Die Kosten für notwendige Folgeuntersuchungen (STI-Screening etc.) sind für die Klient*innen zu hoch	O	O	O	O	O	O	O	O	O	O	O
Kulturelle Probleme halten die Klient*innen von der Einnahme einer PrEP ab	O	O	O	O	O	O	O	O	O	O	O
Die Klient*innen schätzen ihr eigenes Risiko, sich mit HIV zu infizieren als zu gering ein	O	O	O	O	O	O	O	O	O	O	O
Der Zeitaufwand, regelmäßige Arztbesuche und/oder STI-Screenings wahrzunehmen, ist für die Klient*innen zu hoch	O	O	O	O	O	O	O	O	O	O	O

Die Klient*innen haben Schwierigkeiten, einen Arzt/eine Ärztin zu finden, der/die ihnen eine PrEP verordnet	O	O	O	O	O	O	O	O	O	O	O
Die Klient*innen haben Sorgen vor sozialer Stigmatisierung in der peer group	O	O	O	O	O	O	O	O	O	O	O
Die Klient*innen haben zu wenig Informationen über PrEP in Klient*innen-gerechter Sprache	O	O	O	O	O	O	O	O	O	O	O
Den Klient*innen fehlen Informationen zur PrEP in ihrer Muttersprache	O	O	O	O	O	O	O	O	O	O	O

Gibt es weitere relevante Probleme für potenzielle PrEP-Nutzer*innen, die in Ihrer persönlichen Beratungspraxis auftreten?
Diese können Sie uns hier gerne mitteilen... ___________________________________

____

Zwei letzte Fragen:

Welche der folgenden Informations- oder Schulungsmaterialien würden die Beratungen zur PrEP praktikabler machen oder verbessern? (Mehrfachnennung möglich)

O Eine Leitlinie mit übersichtlicher Darstellung von Indikationen, Kontraindikationen und erforderlichen Untersuchungen
O Eine Entscheidungshilfe für Klient*innen, die Informationen zur PrEP in Klient*innen-verständlicher Art darstellt
O Eine Entscheidungshilfe für Klient*innen, die Informationen zur PrEP in unterschiedlichen Sprachen darstellt
O Informationen oder Schulungen für Berater*innen zum Management von PrEP (z.B. erforderliche Untersuchungen etc.)
O Informationen oder Schulungen für Berater*innen zur Identifikation von Klient*innen, die von PrEP profitieren könnten
O Informationen oder Schulungen zum Thema „Mit Klient*innen über Sexualität sprechen“
O Eine App- oder SMS-gestützte Erinnerungsfunktion für PrEP-Nutzer, um deren Adhärenz zu fördern
O Sonstiges: ____________

Gibt es weitere Aspekte oder Probleme, die Sie in Ihrer Beratungstätigkeit zum Thema PrEP beschäftigen? Haben Sie andere Vorschläge, wie die Beratung von Menschen, die von einer PrEP möglicherweise profitieren könnten, verbessert werden kann?
Diese können Sie uns hier gerne mitteilen... __________________

____

Vielen Dank für Ihre Teilnahme an der Erhebung!
